# Author Reply to the Letter to the Editor: Antimicrobial stewardship without infectious disease physician

**DOI:** 10.1002/jgf2.175

**Published:** 2018-05-21

**Authors:** Minoru Murakami, Hirokazu Komatsu

**Affiliations:** ^1^ Department of Nephrology Saku Central Hospital Nagano Japan; ^2^ Department of Community Care Saku Central Hospital Nagano Japan

## Abstract

We were pleased to read the letter to our article and decided to respond to the letter.

To the Editor

We thank the author of the *Letter to the Editor* 1 for his interest in our article published recently in this journal.^2^ First, the author was interested in further information on the prognosis of the five patients of the preintervention group who received no antifungal treatment. Among those five patients, four died by day 30, and therefore, we considered that the results remained unchanged. Although we defined an episode of candidemia as isolation of *Candida* spp. in at least one blood culture in a patient with clinical signs and symptoms of infection, as pointed out by the author of the letter, the only single patient who survived without antifungal treatment could be considered to have had no true candidemia that required treatment. However, the Infectious Disease Society of America (IDSA) guideline emphasizes the importance of addressing a positive blood culture result with prompt initiation of systemic antifungal therapy, because delays in such treatment are associated with increased mortality.^3^ As pointed out by the author of the letter, it was extremely difficult to differentiate *Candida* spp. from contaminants.

Second, the author^1^ pointed out that more discussion was needed regarding the reason for why antimicrobial stewardship team (AST) without an infectious disease physician (IDP) did not improve mortality despite improving adherence to the IDSA guidelines for the management of candidemia. It was difficult to find other reasons apart from the three likely explanations stated in the Discussion section of our paper^2^ because this was the first report to suggest the association between non‐IDP AST and improved adherence to the guidelines in patients with candidemia. As pointed out in the *Letter to the Editor,* 1 the clinical judgment of the IDP is considered the most important aspect of management that can reduce the mortality rate in candidemia. The IDSA guidelines state that adherence to these guidelines is voluntary, with the ultimate determination regarding application to be made by the physician based on the individual circumstances of each patient.^4^ However, it is noteworthy that even IDP‐based AST seems to have limited value in improving prognosis of patients with candidemia.^5^ Further studies are warranted to confirm whether the inclusion of the AST is associated with lower mortality in patients with candidemia even if the team does not include IDPs.

Finally, the author^1^ pointed out that the initial sentence under the Primary Outcome section of the Results section was incorrect. We thank the author for the comment and apologize for the incorrect statement. The text should read as follows: Of the intervention group, 11 of 46 (23.9%) patients died by 30 days, compared with 7 of 30 (23.3%) patients of the preintervention group. Luckily, this error does not affect the results or conclusions of our study.

## CONFLICT OF INTEREST

The authors have stated explicitly that there are no conflicts of interest in connection with this article.
